# Laboratory management for large‐scale population screening for SARS‐CoV‐2

**DOI:** 10.1002/iid3.535

**Published:** 2021-11-22

**Authors:** Xiuzhi Duan, Dingfeng Lv, Lin Wang, Yanchao Liu, Shu Zhang, Weiwei Liu, Jiamin Xie, Chunqiang Gu, Xiaosi Li, Zhihua Tao, Xiang Chen, Qiang Yao

**Affiliations:** ^1^ Department of Laboratory Medicine The Second Affiliated Hospital of Zhejiang University School of Medicine Hangzhou Zhejiang China; ^2^ Department of Blood Transfusion Ningbo First Hospital Ningbo Zhejiang China; ^3^ Department of Laboratory Medicine Zhejiang University Hangzhou Zhejiang China; ^4^ Department of Virology Research Ningbo Municipal Center for Disease Control and Prenvention Ningbo Zhejiang China; ^5^ Department of Laboratory Medicine Sir Run Run Shaw Hospital Hangzhou Zhejiang China; ^6^ Department of Laboratory Medicine, The First Hospital of Jiaxing Affiliated Hospital of Jiaxing University Jiaxing Zhejiang China; ^7^ Department of Laboratory Medicine The Second Affiliated Hospital of Jiaxing University Jiaxing Zhejiang China; ^8^ Medical Administration Office Health Commission of Zhejiang Province Hangzhou Zhejiang China; ^9^ Director's Office Zhejiang Provincial Dermatology Hospital Huzhou Zhejiang China

**Keywords:** laboratory management, mass screening, nucleic acid detection, SARS‐CoV‐2

## Abstract

**Introduction:**

The aim of this study was to investigate the improvements in laboratory testing procedures and the quality and safety management for large‐scale population screening for severe acute respiratory syndrome coronavirus 2 (SARS‐CoV‐2).

**Methods:**

Because of epidemic prevention and control needs in Hebei Province, on January 7, 2021, the Health Commission of Zhejiang Province sent a medical team to Hebei Province, to carry out SARS‐CoV‐2 nucleic acid testing. Screening for the SARS‐CoV‐2 nucleic acid test was performed using reverse‐transcription polymerase chain reaction (RT‐PCR). Practical tests and repeated process optimization were adopted to explore the optimal solution for improving laboratory testing procedures and the quality of and safety management for large‐scale population screening for SARS‐CoV‐2.

**Results:**

The Zhejiang medical team completed 250,000 pooled SARS‐CoV‐2 nucleic acid samples in 24 days in Shijiazhuang, with a peak daily testing capacity of 40,246 samples testing. There were no false‐negative or false‐positive results, and no laboratory personnel was infected with SARS‐CoV‐2. Significant achievements have been made in SARS‐CoV‐2 prevention and control.

**Conclusions:**

This report summarizes the effort of the medical team regarding their management of the quality and safety of laboratory tests and proposes corresponding empirical recommendations to provide a reference for future large‐scale population screening SARS‐CoV‐2.

## Graphical abstract

The Health Commission of Zhejiang Province of china sent a medical team to Hebei Province, to carry out SARS‐CoV‐2 nucleic acid testing. The Zhejiang medical team completed 250,000 pooled more than two million peoples' SARS‐CoV‐2 nucleic acid samples in 24 days in Shijiazhuang, with a peak daily testing volume of 40,246 pooled samples using reverse‐transcription polymerase chain reaction (RT‐PCR). There were no false‐negative or false‐positive results, and no laboratory personnel was infected with SARS‐CoV‐2. Layout recommendations for temporary laboratories for large‐scale nucleic acid screening operation process and experience sharing of each link of nucleic acid detection.

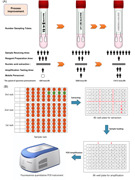

## 1 INTRODUCTION

The spread of the new pathogen severe acute respiratory syndrome coronavirus 2 (SARS‐CoV‐2) has caused an expanding pandemic of coronavirus disease‐2019 (COVID‐19).^1^ As of September 27, 2020, over 32.7 million COVID‐19 cases and 991,000 deaths have been reported to the WHO.^2^ Laboratory viral nucleic acid amplification testing (NAAT), such as real‐time reverse‐transcription polymerase chain reaction (RT‐PCR), has been recommended by the WHO as a standard confirmation protocol for SARS‐CoV‐2 detection.^3^ NAAT shows better performance than immunoassays, as it can identify viral RNA in the early stage of infection. Since 1988, PCR technology has been extensively used in clinical laboratories due to its advantages of high sensitivity and ease of use.^4^ In response to the emergency order issued by the State Council's Joint Medical Team for the Prevention and Control of the COVID‐19 Outbreak in Hebei Province, on January 7, 2021, the Health Commission of Zhejiang Province sent 103 medical personnel and nucleic acid extraction machines, PCR machines, and nucleic acid extraction and amplification reagents, among other materials to Shijiazhuang, Hebei Province. After arrival, 62 personnel remained in Shijiazhuang. In accordance with the “Work Manual for SARS‐CoV‐2 Nucleic Acid Testing in Medical Institutions (Trial Version 2)”,^5^“Medical Treatment Plan for the Response to the COVID‐19 Pandemic in Autumn and Winter,”^6^ and the “Technical Specifications for 10 in 1 Pooled Collection and Detection of SARS‐CoV‐2 Nucleic Acid”^7^ issued by the State Council's Joint Comprehensive Team for the Prevention and Control of the COVID‐19 Outbreak and based on the requirements of the National Health Commission of the People's Republic of China and the experience of the Zhejiang medical team regarding nucleic acid testing in Xinjiang, the testing team developed laboratory job responsibilities, standard operating procedures, and various record forms. This article summarizes and shares the experience and lessons of managing the testing quality and safety of the medical team and provides a reference for possible future large‐scale COVID‐19 population screening.

## 2 MATERIALS AND METHODS

### 2.1 Reagents and consumables

Nucleic acid extraction machines (ZK‐96, Zhongkebio Med Technol), fluorescence quantitative PCR machines (Hongshi SLAN96P), nucleic acid extraction reagents (Wuhan EasyDiagnosis Biomedicine Co., Ltd.), amplification reagents (Wuhan EasyDiagnosis Biomedicine Co., Ltd. [preliminary screening], DAAN Gene Co., Ltd. of Sun Yat‐sen University [retesting], and Sansure Bio‐technology Co., Ltd. [the second re‐testing]), 96‐well plates and parafilm (0.2 ml), biological safety cabinets (numbers: 7), multichannel pipettes, single‐channel pipettes and filter tips of different specifications (10‐µl long pipette tip), eight walkie talkies, and so forth were utilized. The Zhejiang medical team was equipped with 15 nucleic acid extraction machines, 30 PCR machines, and 140,000 bottles of nucleic acid extraction and amplification reagents. During the support period, the Zhejiang medical team received strong support from the First Hospital of Hebei Medical University, which provided various items, instruments, and consumables for the medical team.

### 2.2 Staffing

Sixty‐two personnel composed the testing team, including one team leader, one liaison officer, and six management personnel; the remaining 54 personnel were divided into three groups, with 18 people/groups, covering three 8‐h shifts per day. The initial shifts were later modified to the following times: morning shift, 06:00–15:00; afternoon shift, 15:00–24:00; and night shift, 24:00–06:00. In addition, a total of 32 volunteers from the First Hospital of Hebei Medical University who provided support to the medical team from Zhejiang Province were divided into four groups, with eight people in each group, working 6 h per shift to help with specimen reception and numbering; the volunteers did not conduct SARS‐CoV‐2 nucleic acid testing.

### 2.3 Nucleic acid extraction and fluorescence quantitative PCR method

The virus RNA was extracted using the magnetic beads method, according to the instructions of the nucleic acid extraction kit (Wuhan EasyDiagnosis Biomedicine Co., Ltd.). Nucleic acid extraction needs 30 min according to the instructions and PCR reaction needs 80 min. Each batch requires 1 weak positive quality control product and three normal saline negative control products to participate in the whole process of specimen detection.

The remaining steps in the nucleic acid isolation and real‐time RT‐PCR procedures are unchanged. Following 40 cycles of RT‐PCR, a specimen is deemed positive for SARS‐CoV‐2 RNA when the C_*t*_ values for both targets are <38 cycles. A specimen is deemed negative when the C_*t*_ values for both targets are ≥40 cycles and the internal amplification control C_*t*_ value is <35. Regardless of the result of another target gene, as long as the C_*t*_ value of any target gene is >38, the test result is deemed inconclusive and the specimen is retested. One weak positive and three negative quality control products in each batch are normal. Pooled specimens with negative results and valid internal control results are individually defined as negative. Pooled specimens with positive or inconclusive results or invalid internal control results are repeated as individual specimens to determine the final result for each.

## 3 RESULTS

### 3.1 Establishment and improvement of the laboratory management system

**Figure 1:**
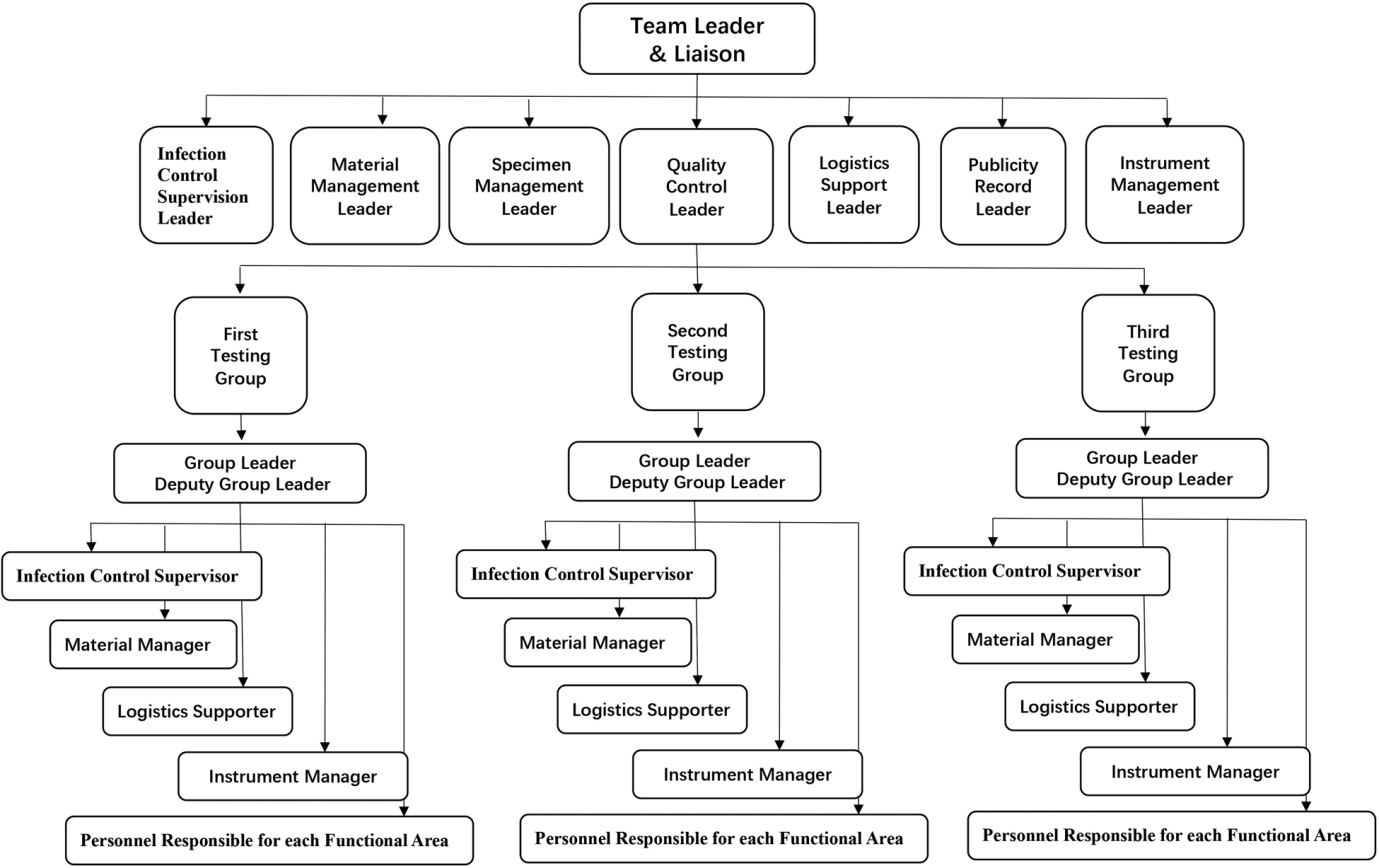
Organizational framework of the testing team. District I: reagent preparation area. District II: sample processing area. District III: polymerase chain reaction amplification area

The medical team arrived in Shijiazhuang, Hebei Province, at 11:00 p.m. The team leader quickly organized the experienced personnel at the First Hospital of Hebei Medical University to conduct a laboratory tour, modify the site, propose laboratory improvement suggestions, and request items that were needed. In the early morning of the next day, a meeting with all personnel was held to determine who would be management personnel on the testing team, establish an organizational framework, and determine who would be members of the management team and who would be the testing team leader and deputy team leader. Six members composed the management group: quality control leader, infection control supervision leader, logistics support leader, specimen management leader, material management leader, and publicity record leader. Fifty‐four testing personnel were divided into three groups, with each group comprising 18 people, including one group leader and one deputy group leader. Each testing team quickly established functional positions for each member. The specific organizational framework for the testing team is shown in Figure 1. After the organization framework was determined, the management personnel quickly developed an initial management system document and conducted a DingTalk conference to train all staff. The quality control team quickly completed the performance evaluation of the instruments and reagents in the new laboratory to confirm the effectiveness of the testing system. The management team quickly formulated relevant documents. The documents were divided into three parts. The first part was the personnel responsibilities for each management leader, the second part was the specific operation process for each operation and emergency incident response operating procedure, and the third part was the records of SARS‐CoV‐2 testing processes, instrument maintenance, contamination monitoring, and positive result retest summary.

### 3.2 Layout and construction of the Shijiazhuang nucleic acid testing laboratory

**Figure 2:**
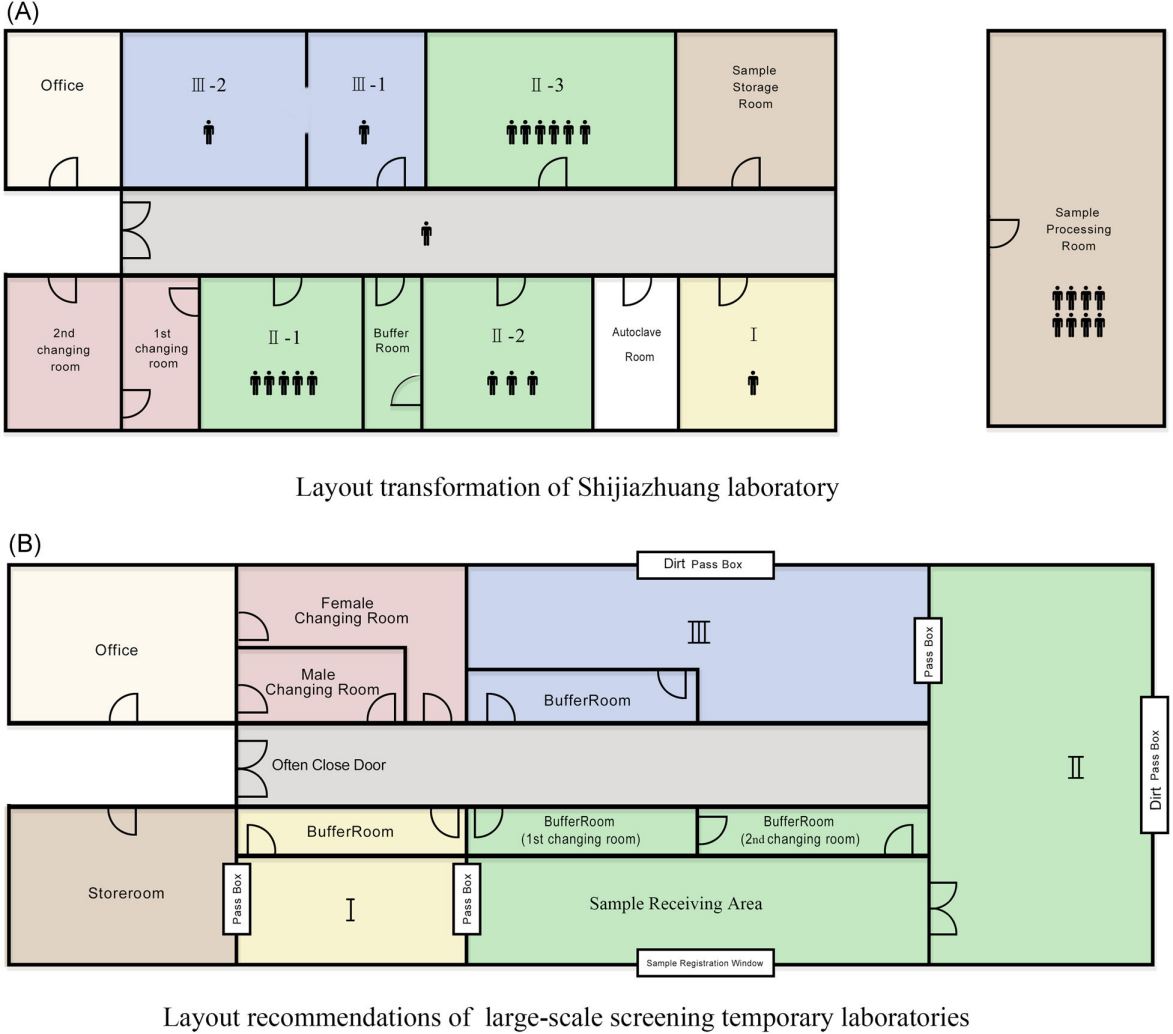
Laboratory division. (A) Layout transformation of Shijiazhuang nucleic acid testing laboratory. (B) Layout recommendations for temporary laboratories for large‐scale nucleic acid screening

Layout transformation of Shijiazhuang laboratory: The testing team modified the layout of the laboratory on the first floor of the First Hospital of Hebei Medical University based on needs, adding a new changing room, a second changing room, an PCR amplification area, an office, a waste corridor, and a sample receiving and processing room. The advantage of this layout is that amplification occurs in one room, facilitating the overall planning and effective use of amplification equipment. The disadvantage is that the sample processing area is divided into three rooms, causing certain barriers to personnel communication and partially reducing the use efficiency of the extraction equipment. See Figure 2A.

Layout recommendations for temporary laboratories for large‐scale nucleic acid screening: After 24 days of workflow optimization, to meet the needs of large‐scale nucleic acid screening, the testing team established a layout for the temporary laboratory that allowed a maximum of 30,000 pooled samples testing per day. Details are provided in Figure 2. (1) The preprocessing area for sample reception must be larger than 50 m^2^. The design of the preprocessing room and the extraction area next door can reduce the sample transfer time and facilitate communication and donning and removing protective clothing. (2) The nucleic acid extraction area and the amplification area should be rooms with sufficiently large areas. To prevent contamination, the amplification area contains a waste outlet for the timely output of PCR products. (3) Dedicating space for an autoclave room inside the laboratory is not recommended. See Figure 2B.

### 3.3 Preprocessing and management of specimens

Sample preprocessing planning and process optimization: Based on a workload of 30,000 pooled samples testing, the Zhejiang medical team was equipped with corresponding processing sites, specimen preprocessing personnel, corresponding specimen mixers, an adequate number of sample racks, and barcode printing paper for serial numbers. The sample preprocessing room selected was approximately 50 m^2^, equipped with a biosafety cabinet and 10 mini‐oscillators. There was a total of 32 volunteers worked for preprocessing and management of specimens. The sample racks were arranged in a row of eight, corresponding to the positions of a 96‐well extraction plate and a 96‐well amplification plate. Each batch requires three negative and one weakly positive quality control samples. Weakly positive quality control sample is in the virus concentration range of 1.5–3 times the minimum detection limit of the amplification reagent. Weakly positive quality control samples were saline that had been placed in a biosafety cabinet for more than 24 h with the lid open to monitor environmental contamination.

**Figure 3:**
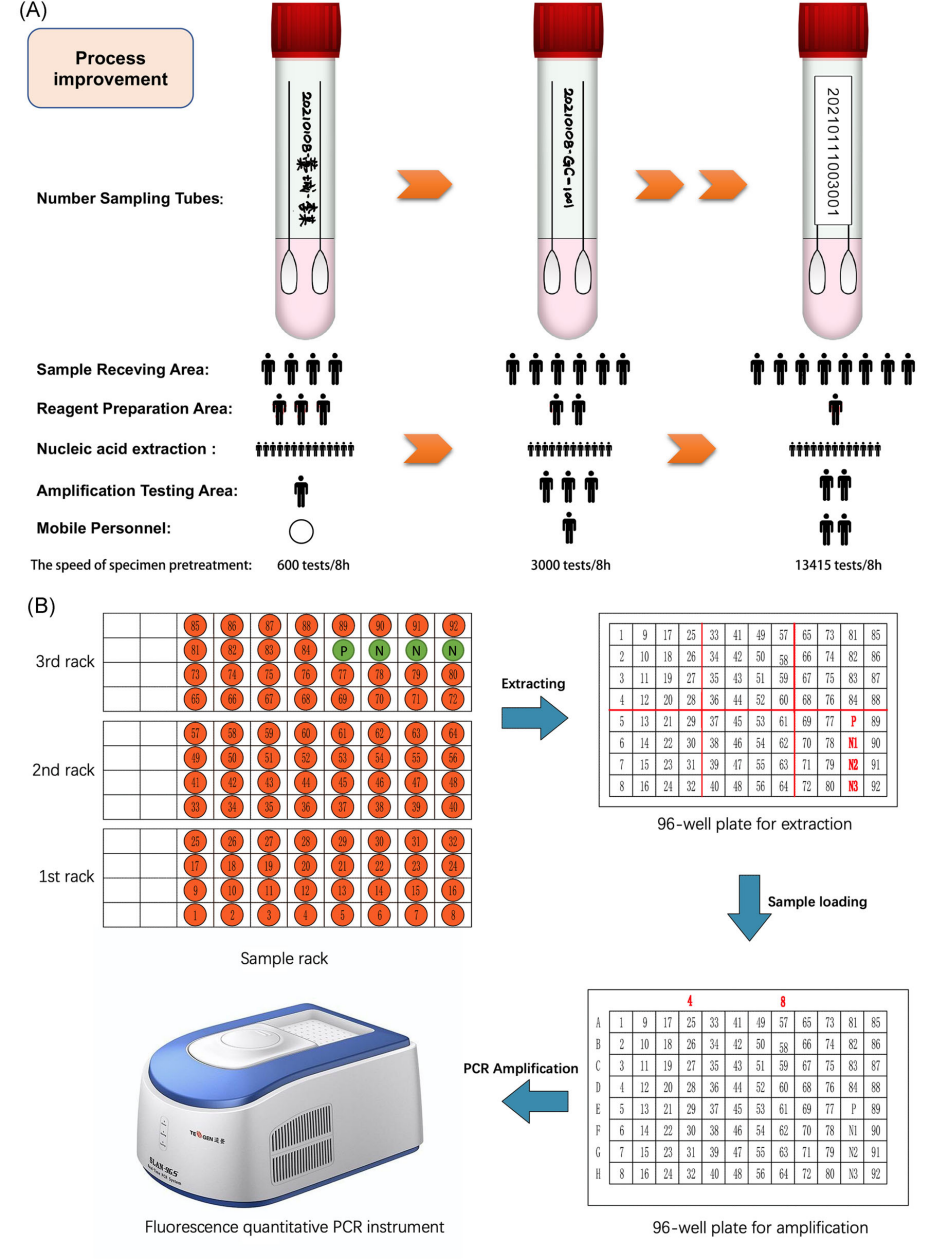
Process improvement and sample processing process. (A) Process improvement, the upper part of picture A is to improve the sampling tube numbering process, and the lower part is to improve the number of personnel in different areas. (B) The position of a sample on the racks and sample processing process

Double crosses were marked on extraction plates to avoid loading errors. The samples were numbered using a barcode printer. After a sample was fully shaken and mixed, the sample was placed into the designated position of the sample rack (the sample numbering standard was 14 digits [date + sample number, e.g., 20210112003001]), and every 100 numbers were a unit (including 92 specimens, 1 weak positive quality control sample, and 3 negative quality control samples, for a total of 96 wells; discard **93‐**100, such as 3001‐3092, discard 3093‐3100). Date and year were removed from the final sample number, and only 5 digits were retained, such as “03069”. The sample racks consisted of 4 rows of 40 samples. The three sample racks were equipped with a “nucleic acid extraction record sheet” and passed to the coordinator in the testing area. The position of a sample on the racks and sample processing process in Figure 3. In 24‐h period, the maximum number of tubes the testing team processed was 40,246, approximately 200 tubes/(person/h); the maximum processing speed can reach 338 tubes/(person/h).

### 3.4 Nucleic acid testing management

#### 3.4.1 Experience in reagent configuration

One person was in the reagent preparation room. The operator configures the PCR mixture according to the reagent operation manual to estimate the number of detections, mix the PCR solution and Taq DNA polymerase, reconstitute the reagent at room temperature, mix and centrifuge, pour them into a disposable sample tank, and draw PCR mixture with 12‐channel pipette to 96‐well plate. After aliquoting the reagents into the plate system, the plate was sealed in a disposable Ziplock bag and stored at room temperature, and the aliquoting time was indicated. The individual who prepared the reagents removed reagents from the freezer before leaving the laboratory for the next shift and placed them at room temperature to thaw. Based on previous experience, the reagent preparation speed was calculated to be 4000 tests/(person/h), and all the systems required for 13,000 pooled samples testing per shift could be prepared in approximately 4 h.

#### 3.4.2 Experience in RNA extraction

The testing team had a total of 15 extraction instruments in three sample processing areas. There were 14 people working during each shift, and each of the three sample extraction rooms had a manager. The extraction personnel was assigned based on the number of safety cabinets, and a separate safety cabinet was used as the fixed location for loading the RNA template. One person was responsible for sample distribution, extraction, medical waste collection, and template transfer. After loading the samples and template, the individual completed the “Nucleic Acid Extraction Record Form.” To better trace samples, untested samples were placed at the "untested sample" location and were covered by small yellow garbage bags. After sample loading was complete, the samples and sample racks covered with the small yellow garbage bags were placed at the “tested samples” location. The yellow garbage bags followed the specimens until the final report for review. Then, the samples were removed from the racks and sealed in the small yellow bags, which were then placed into a large yellow garbage bag. A medical waste label was put on the outer surface of the bag, the date and number range were written on the bag, and the bag was stored in a designated location. The above process created efficiency for sample retesting. It took approximately 15–20 min for a single person to add samples into 96 wells with a single‐channel pipette, that is, a single person can load approximately 276–368 tubes/(person/h).

#### 3.4.3 Experience in PCR amplification testing

District III was approximately 40 m^2^, containing a total of 30 PCR instruments. Each shift had two experienced team members for plate loading and result auditing. The personnel from District III analyzed the test results and completed the “Nucleic Acid Extraction Record Form” and the “Handover Form for Sample Transfer from District III to District II.” Regarding retesting criteria, the personnel in District III provided the sample number for abnormal results in the "Handover Form for Sample Transfer from District III to District II" and returned the sample to District II; samples with unqualified internal reference curves or low/flat amplification curves were retested, and suspected positive samples were reextracted using duplicate wells and simultaneously amplified using duplicate wells. The computed tomography (CT) values for positive results were registered in the “Nucleic Acid Extraction Record Form.” The testing team in Shijiazhuang reached the daily test capacity of 40,246 pooled samples.

#### 3.4.4 Mass screening laboratory quality control essentials

(1) Return of unqualified specimens: Samples tested twice without internal reference gene; samples without sampling liquid or sampling brush; leakage of sampling fluid; sample barcode cannot be identified; sampling liquid does not match the PCR detection system.
(2) Quality control of sample extraction: Three racks with samples were placed in bag with an original record as one unit to facilitate specimen search and retest in the later.
(3) Retest rules: (1) Retest the original sample using the screening reagent (EasyDiagnosis): Reference gene CT > 35 or ORF1a/B or N gene showed atypical S‐shaped curve; (2) retest five samples using two reagents (EasyDiagnosis and DaAn): When a specimen is found to have a typical S‐shaped curve in ORF1a/b and N genes, retest the abnormal specimen and its upper and lower, left and right, a total of five samples. (3) Retest the whole unit using the screening reagent (EasyDiagnosis): The quality control result of this unit was error; the retest result of the positive sample was inconsistent with the first result.

### 3.5 Laboratory safety management

In accordance with the “Work Manual for SARS‐CoV‐2 Nucleic Acid Testing in Medical Institutions (Trial Version 2)”^5^ and “Medical Treatment Plan for the Response to the COVID‐19 Pandemic in Autumn and Winter”^6^ issued by the State Council's Joint Comprehensive Team for the Prevention and Control of the COVID‐19 Outbreak, we used the inactivated virus sample tubes (such as including guanidine salt or surfactant), nucleic acid detection in biological safety laboratory for Class 2, take the appropriate individual protection measures. At the same time, we also carried out the following protective measures: (1) Regarding the safety of the testing process, the testing team strictly implemented the personal protection standards of the laboratory, supervised the donning and removal of protective equipment such as protective clothing, and strictly disinfected the personnel and objects after each shift. Microbial cultures of high‐frequency contact surfaces in the laboratory and SARS‐CoV‐2 nucleic acid testing of the surfaces of key objects were conducted every 3 days. (2) Regarding logistics, centralized dining was replaced with a self‐pickup method, a buffer room was set up for team members in the hotel to disinfect and change clothes when going to and from the hospital, commuter vehicles were regularly disinfected, and on‐site meetings were replaced with DingTalk. (3) Regarding the safety of the team members, fire‐fighting training, nosocomial infection management training, and daily body temperature monitoring were conducted, and all personnel conducted nucleic acid self‐tests every 3 days. The physical and mental health of medical team members was prioritized, and unwell team members were replaced in a timely manner if needed.

### 3.6 Recommend appropriate personnel and equipment ratios of large‐scale SARS‐CoV‐2 nucleic acid screening

#### 3.6.1 The maximum daily testing capacity recommended by the National Health Commission of China

The National Health Commission proposed^8^ guidelines for the configuration of testing personnel based on a daily testing capacity of 10,000 pooled samples: 10–12 amplification instruments, six 96‐well extraction instruments, 24–25 testing personnel, and 15 auxiliary personnel. Based on an amplification and extraction speed of 30 min/batch, each PCR instrument can complete 833–1000 pooled samples/day, each extraction instrument can extract 1667–2500 pooled samples/day, and the testing capacity per person is 400–417 pooled samples/day. The formula for calculating the maximum equipment‐to‐person ratio recommended by the National Health Commission is *N* = 1000 × *A* = 2500 × *B* = 417 × *C* (*N*: number of samples tested per day, *A*: total number of PCR instruments, *B*: total number of extraction instruments, and *C*: total number of people, Table 1).

**Table 1:** Results of the maximum testing capacity of the large‐scale SARS‐CoV‐2 nucleic acid screening (amplification time: 90 min, extraction time: 30 min)

Classification	PCR instrument (test/unit)	Extraction instrument (test/set)	Personnel (test/person)	Equipment to personnel ratio
The maximum daily testing capacity recommended by the National Health Commission	1000	2000	417	*N* = 1000 × *A* = 2500 × *B* = 417 × *C*
The actual maximum daily testing speed in Shijiazhuang	1341	2683	649	*N* = 1341 × *A* = 2683 × *B* = 649 × *C*

#### 3.6.2 Maximum daily testing capacity in actual operation

The testing team had a total of 30 PCR machines, 15 nucleic acid extraction instruments, and 62 testing personnel. Based on the actual operation of 40,246 samples testing per day, the maximum daily testing capacity per person was 649 pooled samples. The actual optimization of the equipment to personnel ratio is *N* = 1341 × *A* = 2683 × *B* = 649 × *C* (*N*: number of samples tested per day, *A*: total number of PCR instruments, *B*: total number of extraction instruments, and *C*: total number of personnel).

## 4 DISCUSSION

During the aid to Shijiazhuang City, Hebei Province, the Zhejiang medical team tested 250,000 SARS‐CoV‐2 nucleic acid tests including 10 positive samples, with a maximum daily detection volume of 40,246. The team provided five external quality assessment samples for assessment by the Hebei Clinical Laboratory Center; helped local testing institutions review five suspicious positive samples; and compiled the “Nucleic Acid Testing Handbook for the Hebei Medical Team.” In the late stages at Shijiazhuang, after several process optimization and rectification cycles, the testing team almost reached the maximum theoretical daily testing capacity.

During sample preprocessing, because there was no unified identity entry system for sample collection during the early stage of the COVID‐19 outbreak in Hebei, internal sample numbering by the laboratory was critical for testing and for identifying suspicious samples. With the absence of barcodes, the medical team used machine‐based numbering, which played a significant role in the effective management of specimens. During the reagent preparation process in District I, at the initial stage, one person from District III was assigned to assist District I for 1 h, and the personnel in District I responsible for reagent preparation helped other members after preparing all reagents for the shift. The work conducted in District II involved the most manual steps in the entire nucleic acid testing process. Therefore, the positioning of various items in District II and the positioning of the personnel in this district and an assembly line operation were particularly important to avoid physical exhaustion and time wasting caused by walking back and forth. Every set of 96 specimens was placed in a small yellow garbage bag, and the range of specimen numbers was marked on the opening of the bag, saving considerable time for subsequent specimen review and tracing. Two‐person verification was utilized to effectively reduce the misjudgment rate during results auditing in District III.

In the process of improving the management system, the quality control team conducted daily audits of the initial laboratory results and quality control conditions, randomly checked the quality control systems, and calculated the interbatch deviations; tracked abnormal results, found the source, and implemented retesting; and inspected and supervised reagent preparation, sample extraction, and system amplification steps daily. The laboratory quality management system was continuously modified and improved based on various conditions; in addition, each test shift included 1 h for maintenance and disinfection of extraction equipment and removal of medical waste, effectively reducing the handover time to different shifts and avoiding equipment failures, laying a solid foundation for the successful completion of the next shift.

During the assistance period, all the team members were in good health, no work stoppage occurred, no one was infected with COVID‐19, and the support mission was successfully completed. The daily test rate of 1000 pooled samples/instrument proposed by the National Health Commission was surpassed (actual 1341 tubes), and the daily workload of routine laboratories was increased from 400 pooled samples/person to approximately 649 pooled samples/person at last. Improvements in these data are a result not only of the quality of the testing personnel but also of a rigorous review of the whole laboratory testing process.

There are also shortcomings in the process of this study. The speed of collecting nasopharyngeal swabs during the nucleic acid testing process directly affects the later laboratory testing speed. The failure to use barcode numbers in the presampling of laboratories caused certain problems in the identification and traceability of laboratory samples. In addition, because many brands of sampling tubes are used in large‐scale screening work, some of the sampling tubes of some brands have not been verified for the matching degree of nucleic acid extraction. As a result, the residual PCR inhibitory components in the nucleic acid extract of some sampling tubes affect the follow‐up amplification analysis, this situation may cause some low‐concentration SARS‐CoV‐2 infections to be missed. Therefore, the authors believe that it is important to develop a set of sample collection software that can generate a patient unique identification number that is compatible with laboratory software systems and automatically numbered, which can greatly save the time for numbering specimens and the delivery of patient reports. Second, manufacturers of large‐scale extraction reagents and detection reagents should verify the matching of sampling tubes, and the reagent instructions should recommend brands or main ingredients.

## CONFLICT OF INTERESTS

The authors declare that the submitted work was carried out in the absence of any personal, professional, or financial relationships that could potentially be construed as a conflict of interest.

## AUTHOR CONTRIBUTIONS

Qiang Yao, as the general director of the Zhejiang nucleic acid testing medical team, directed and coordinated all the work. Xiang Chen, as the direct contact person of Zhejiang Provincial Health Commission, was responsible for material distribution and personnel dispatch. Zhihua Tao is the support personnel of quality management. Xiuzhi Duan was the quality management leader of the team, working on process improvement and article writing. Dingfeng Lv as the deputy leader of quality management, to assist in process improvement. Lin Wang, as the group leader of sample preprocessing, improved the process of sample preprocessing. Yanchao Liu, Shu Zhang, and Weiwei Liu, as the leaders of each test group, carried out the specific test work and coordinated the team members. Jiamin Xie and Chunqiang Gu, as the deputy leaders of each testing group, carried out specific testing and assisted the leaders. Xiaosi Li, as the team leader of instrument management, is responsible for instrument maintenance.
